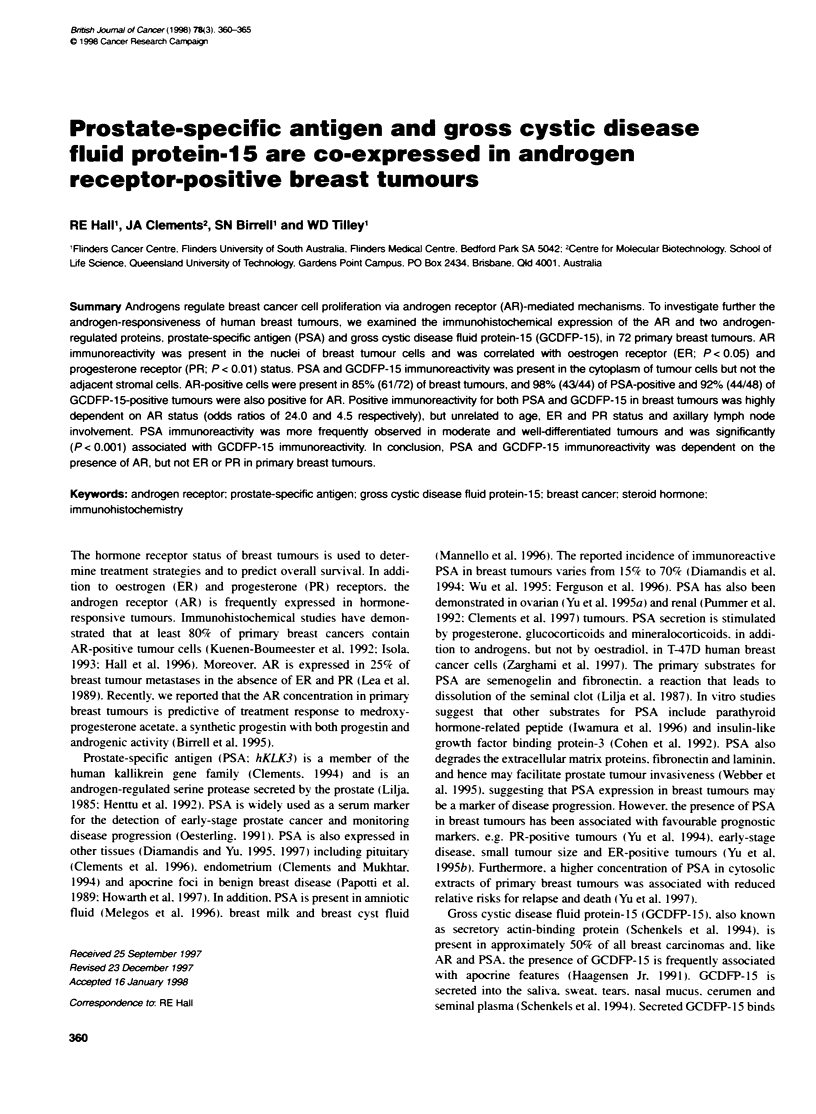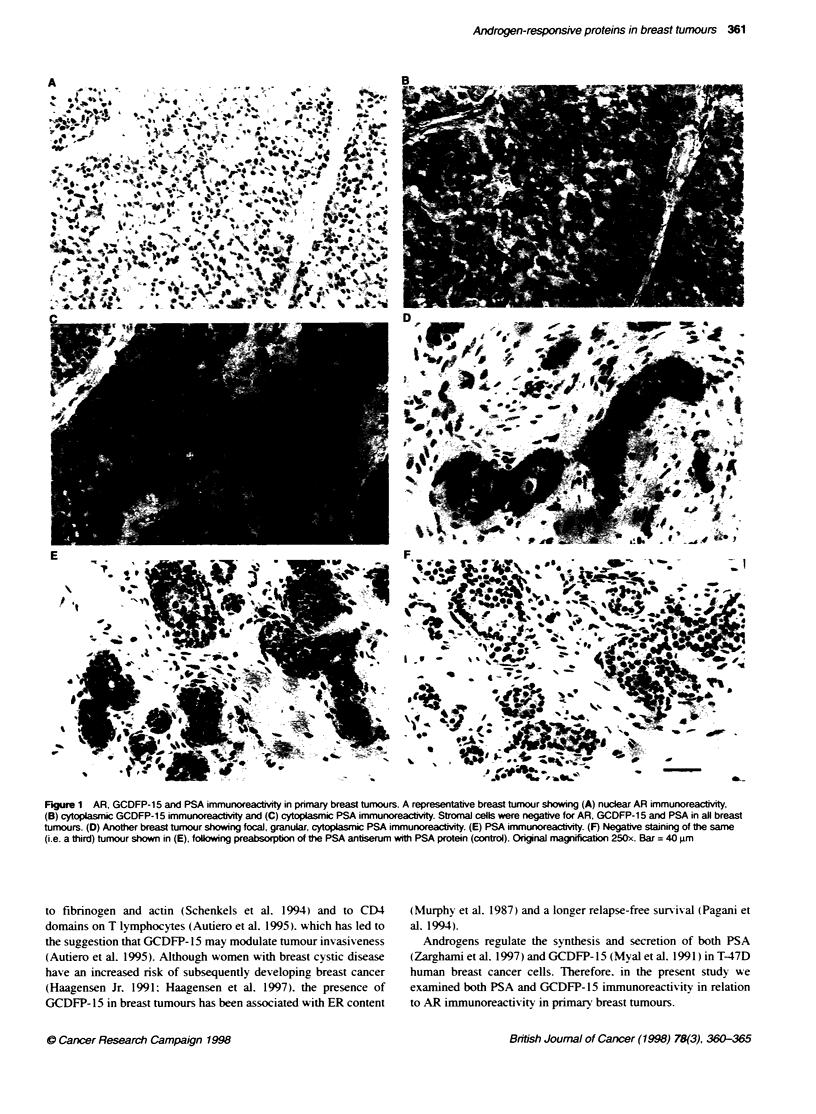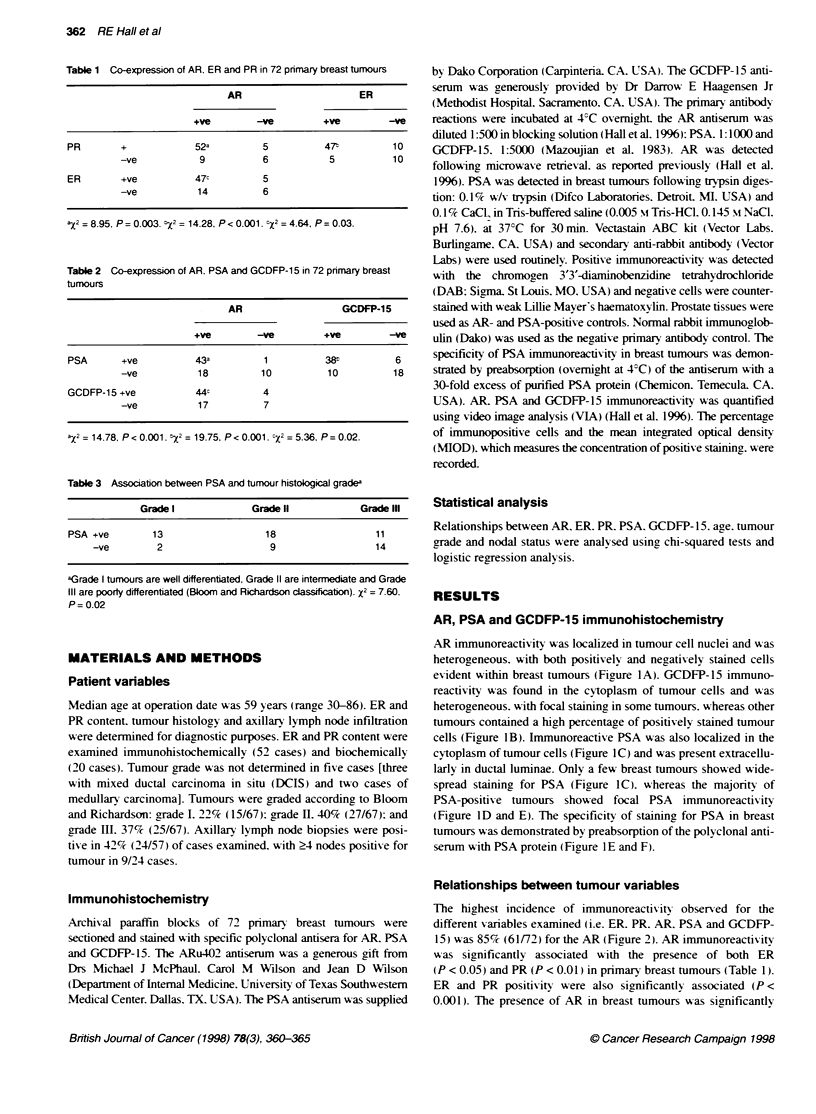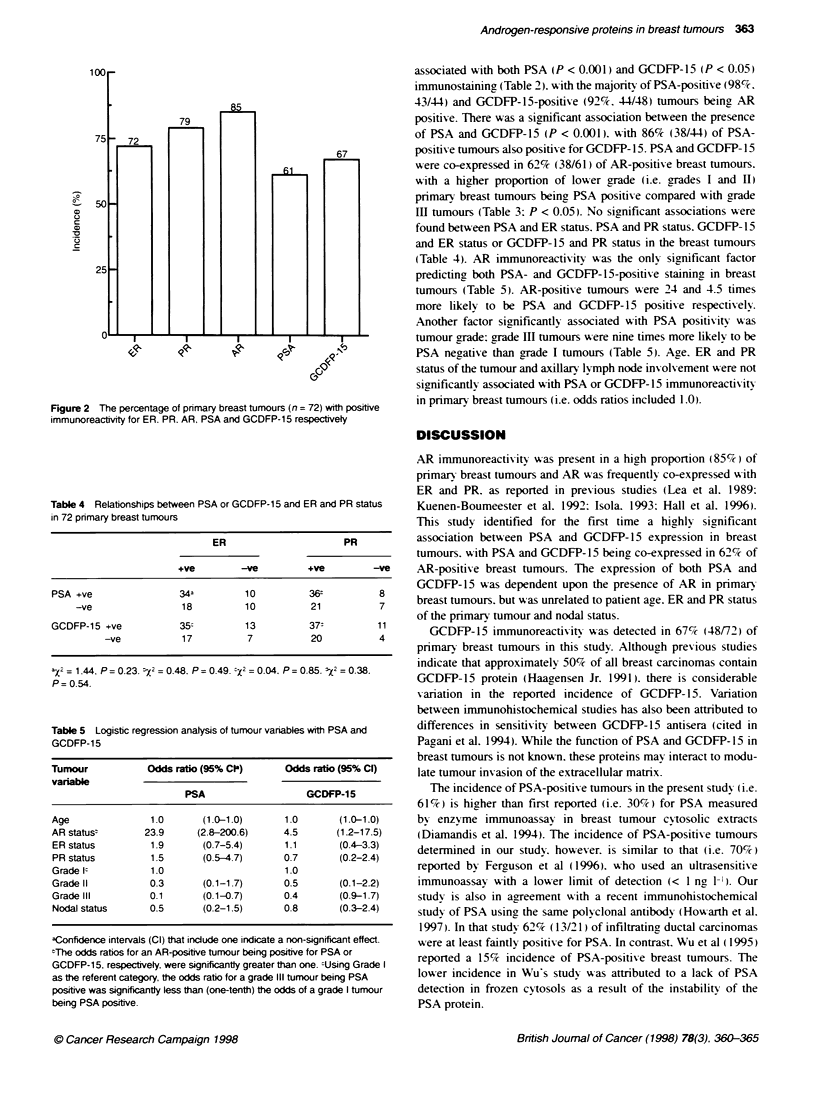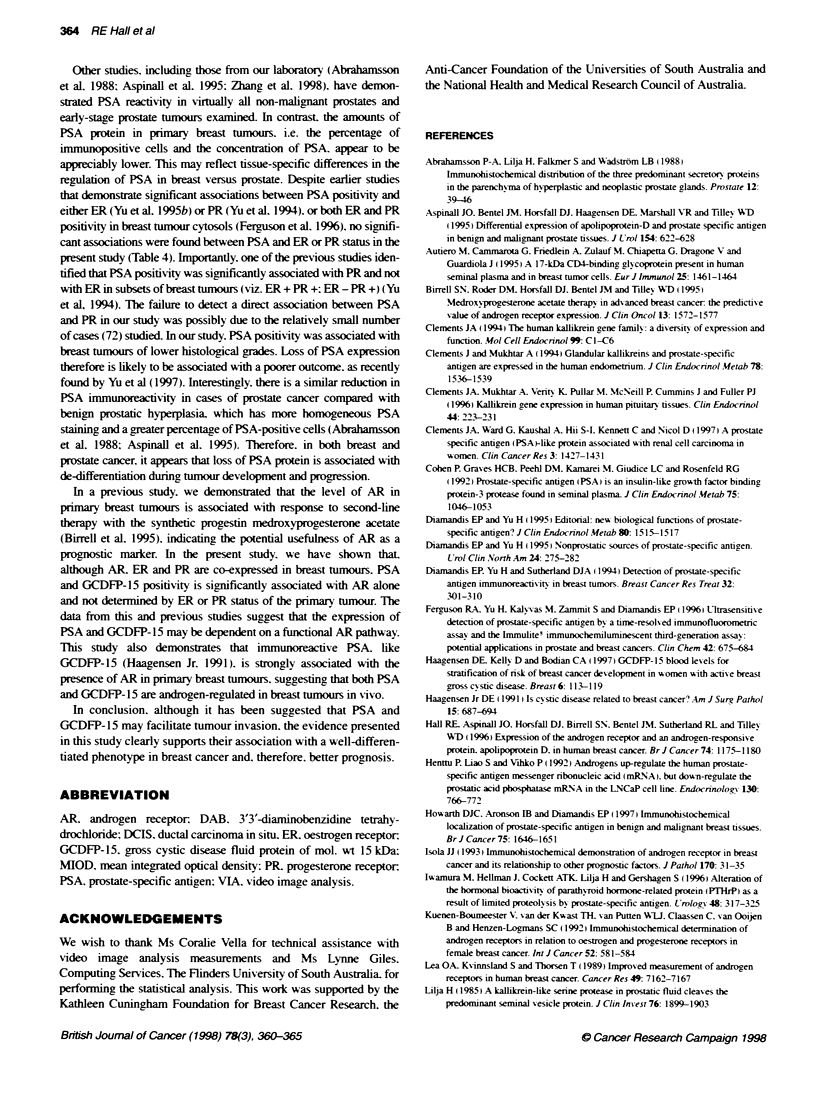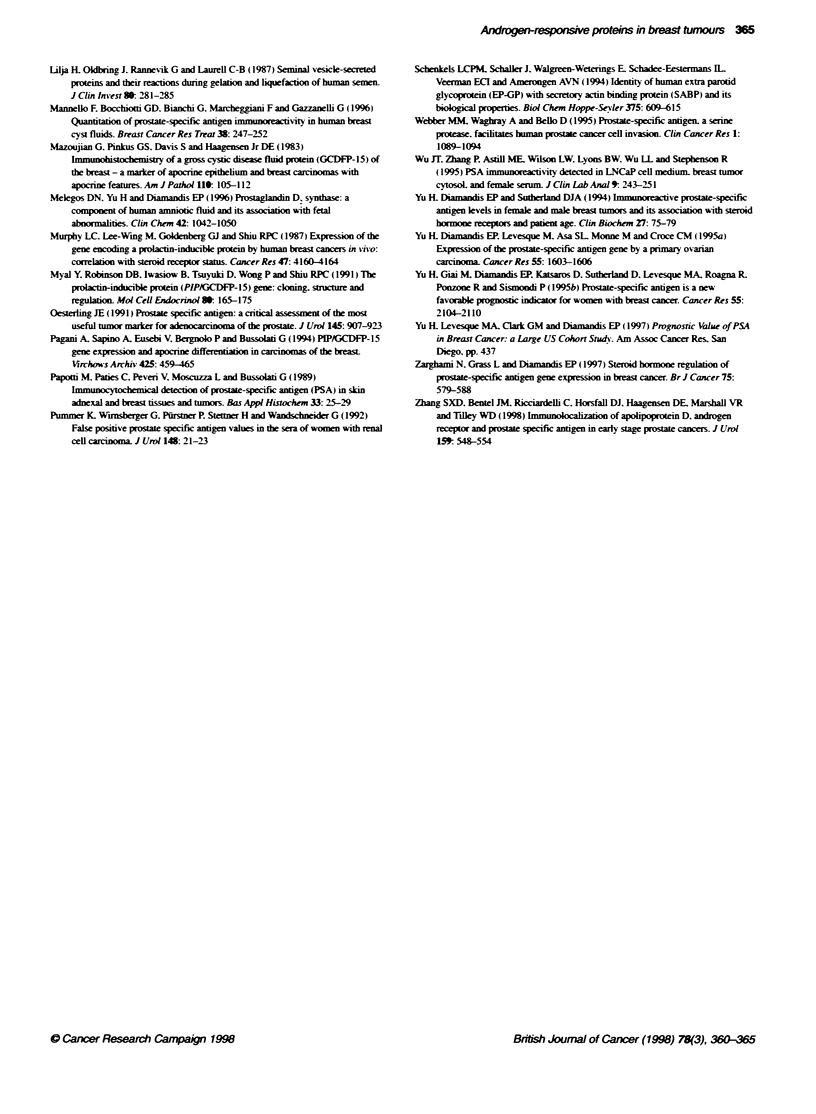# Prostate-specific antigen and gross cystic disease fluid protein-15 are co-expressed in androgen receptor-positive breast tumours.

**DOI:** 10.1038/bjc.1998.499

**Published:** 1998-08

**Authors:** R. E. Hall, J. A. Clements, S. N. Birrell, W. D. Tilley

**Affiliations:** Flinders Cancer Centre, Flinders University of South Australia, Flinders Medical Centre, Bedford Park, Australia.

## Abstract

**Images:**


British Journal of Cancer (1998) 78(3). 360–365
© 1998 Cancer Research Campaign

### Prostate-specific antigen and gross cystic disease fluid protein-15 are co-expressed in androgen receptor-positive breast tumours

**RE Hall[1], JA Clements[2], SN Birrell[1] and WD Tilley[1]**

[1]Flinders Cancer Centre, Flinders University of South Australia, Flinders Medical Centre, Bedford Park SA 5042; [2]Centre for Molecular Biotechnology, School of Life Science, Queensland University of Technology, Gardens Point Campus, PO Box 2434, Brisbane, Qld 4001, Australia

**Summary** Androgens regulate breast cancer cell proliferation via androgen receptor (AR)-mediated mechanisms. To investigate further the androgen-responsiveness of human breast tumours, we examined the immunohistochemical expression of the AR and two androgen-regulated proteins, prostate-specific antigen (PSA) and gross cystic disease fluid protein-15 (GCDFP-15), in 72 primary breast tumours. AR immunoreactivity was present in the nuclei of breast tumour cells and was correlated with oestrogen receptor (ER; $P < 0.05$) and progesterone receptor (PR; $P < 0.01$) status. PSA and GCDFP-15 immunoreactivity was present in the cytoplasm of tumour cells but not the adjacent stromal cells. AR-positive cells were present in 85% (61/72) of breast tumours, and 98% (43/44) of PSA-positive and 92% (44/48) of GCDFP-15-positive tumours were also positive for AR. Positive immunoreactivity for both PSA and GCDFP-15 in breast tumours was highly dependent on AR status (odds ratios of 24.0 and 4.5 respectively), but unrelated to age, ER and PR status and axillary lymph node involvement. PSA immunoreactivity was more frequently observed in moderate and well-differentiated tumours and was significantly ($P < 0.001$) associated with GCDFP-15 immunoreactivity. In conclusion, PSA and GCDFP-15 immunoreactivity was dependent on the presence of AR, but not ER or PR in primary breast tumours.

**Keywords:** androgen receptor; prostate-specific antigen; gross cystic disease fluid protein-15; breast cancer; steroid hormone; immunohistochemistry

The hormone receptor status of breast tumours is used to determine treatment strategies and to predict overall survival. In addition to oestrogen (ER) and progesterone (PR) receptors, the androgen receptor (AR) is frequently expressed in hormone-responsive tumours. Immunohistochemical studies have demonstrated that at least 80% of primary breast cancers contain AR-positive tumour cells (Kuenen-Boumeester et al, 1992; Isola, 1993; Hall et al, 1996). Moreover, AR is expressed in 25% of breast tumour metastases in the absence of ER and PR (Lea et al, 1989). Recently, we reported that the AR concentration in primary breast tumours is predictive of treatment response to medroxyprogesterone acetate, a synthetic progestin with both progestin and androgenic activity (Birrell et al, 1995).

Prostate-specific antigen (PSA; *hKLK3*) is a member of the human kallikrein gene family (Clements, 1994) and is an androgen-regulated serine protease secreted by the prostate (Lilja, 1985; Henttu et al, 1992). PSA is widely used as a serum marker for the detection of early-stage prostate cancer and monitoring disease progression (Oesterling, 1991). PSA is also expressed in other tissues (Diamandis and Yu, 1995, 1997) including pituitary (Clements et al, 1996), endometrium (Clements and Mukhtar, 1994) and apocrine foci in benign breast disease (Papotti et al, 1989; Howarth et al, 1997). In addition, PSA is present in amniotic fluid (Melegos et al, 1996), breast milk and breast cyst fluid (Mannello et al, 1996). The reported incidence of immunoreactive PSA in breast tumours varies from 15% to 70% (Diamandis et al, 1994; Wu et al, 1995; Ferguson et al, 1996). PSA has also been demonstrated in ovarian (Yu et al, 1995*a*) and renal (Pummer et al, 1992; Clements et al, 1997) tumours. PSA secretion is stimulated by progesterone, glucocorticoids and mineralocorticoids, in addition to androgens, but not by oestradiol, in T-47D human breast cancer cells (Zarghami et al, 1997). The primary substrates for PSA are semenogelin and fibronectin, a reaction that leads to dissolution of the seminal clot (Lilja et al, 1987). In vitro studies suggest that other substrates for PSA include parathyroid hormone-related peptide (Iwamura et al, 1996) and insulin-like growth factor binding protein-3 (Cohen et al, 1992). PSA also degrades the extracellular matrix proteins, fibronectin and laminin, and hence may facilitate prostate tumour invasiveness (Webber et al, 1995), suggesting that PSA expression in breast tumours may be a marker of disease progression. However, the presence of PSA in breast tumours has been associated with favourable prognostic markers, e.g. PR-positive tumours (Yu et al, 1994), early-stage disease, small tumour size and ER-positive tumours (Yu et al, 1995*b*). Furthermore, a higher concentration of PSA in cytosolic extracts of primary breast tumours was associated with reduced relative risks for relapse and death (Yu et al, 1997).

Gross cystic disease fluid protein-15 (GCDFP-15), also known as secretory actin-binding protein (Schenkels et al, 1994), is present in approximately 50% of all breast carcinomas and, like AR and PSA, the presence of GCDFP-15 is frequently associated with apocrine features (Haagensen Jr, 1991). GCDFP-15 is secreted into the saliva, sweat, tears, nasal mucus, cerumen and seminal plasma (Schenkels et al, 1994). Secreted GCDFP-15 binds

*Received 25 September 1997*
*Revised 23 December 1997*
*Accepted 16 January 1998*

*Correspondence to:* RE Hall

**Figure 1** AR, GCDFP-15 and PSA immunoreactivity in primary breast tumours. A representative breast tumour showing (**A**) nuclear AR immunoreactivity, (**B**) cytoplasmic GCDFP-15 immunoreactivity and (**C**) cytoplasmic PSA immunoreactivity. Stromal cells were negative for AR, GCDFP-15 and PSA in all breast tumours. (**D**) Another breast tumour showing focal, granular, cytoplasmic PSA immunoreactivity. (**E**) PSA immunoreactivity. (**F**) Negative staining of the same (i.e. a third) tumour shown in (**E**), following preabsorption of the PSA antiserum with PSA protein (control). Original magnification 250×. Bar = 40 μm to fibrinogen and actin (Schenkels et al. 1994) and to CD4 domains on T lymphocytes (Autiero et al. 1995), which has led to the suggestion that GCDFP-15 may modulate tumour invasiveness (Autiero et al. 1995). Although women with breast cystic disease have an increased risk of subsequently developing breast cancer (Haagensen Jr. 1991; Haagensen et al. 1997), the presence of GCDFP-15 in breast tumours has been associated with ER content (Murphy et al. 1987) and a longer relapse-free survival (Pagani et al. 1994).

Androgens regulate the synthesis and secretion of both PSA (Zarghami et al. 1997) and GCDFP-15 (Myal et al. 1991) in T-47D human breast cancer cells. Therefore, in the present study we examined both PSA and GCDFP-15 immunoreactivity in relation to AR immunoreactivity in primary breast tumours.

*© Cancer Research Campaign 1998* 

Table 1 Co-expression of AR. ER and PR in 72 primary breast tumours

| | | AR | | ER | |
|---|---|---|---|---|---|
| | | +ve | −ve | +ve | −ve |
| PR | + | 52[a] | 5 | 47[b] | 10 |
| | −ve | 9 | 6 | 5 | 10 |
| ER | +ve | 47[c] | 5 | | |
| | −ve | 14 | 6 | | |

[a]$\chi^2$ = 8.95. $P$ = 0.003. [b]$\chi^2$ = 14.28. $P$ < 0.001. [c]$\chi^2$ = 4.64. $P$ = 0.03.

Table 2 Co-expression of AR. PSA and GCDFP-15 in 72 primary breast tumours

| | | AR | | GCDFP-15 | |
|---|---|---|---|---|---|
| | | +ve | −ve | +ve | −ve |
| PSA | +ve | 43[a] | 1 | 38[b] | 6 |
| | −ve | 18 | 10 | 10 | 18 |
| GCDFP-15 | +ve | 44[c] | 4 | | |
| | −ve | 17 | 7 | | |

[a]$\chi^2$ = 14.78. $P$ < 0.001. [b]$\chi^2$ = 19.75. $P$ < 0.001. [c]$\chi^2$ = 5.36. $P$ = 0.02.

Table 3 Association between PSA and tumour histological grade[a]

| | | Grade I | Grade II | Grade III |
|---|---|---|---|---|
| PSA | +ve | 13 | 18 | 11 |
| | −ve | 2 | 9 | 14 |

[a]Grade I tumours are well differentiated. Grade II are intermediate and Grade III are poorly differentiated (Bloom and Richardson classification). $\chi^2$ = 7.60. $P$ = 0.02

### MATERIALS AND METHODS

### Patient variables

Median age at operation date was 59 years (range 30–86). ER and PR content. tumour histology and axillary lymph node infiltration were determined for diagnostic purposes. ER and PR content were examined immunohistochemically (52 cases) and biochemically (20 cases). Tumour grade was not determined in five cases [three with mixed ductal carcinoma in situ (DCIS) and two cases of medullary carcinoma]. Tumours were graded according to Bloom and Richardson: grade I. 22% (15/67): grade II. 40% (27/67): and grade III. 37% (25/67). Axillary lymph node biopsies were positive in 42% (24/57) of cases examined. with ≥4 nodes positive for tumour in 9/24 cases.

### Immunohistochemistry

Archival paraffin blocks of 72 primary breast tumours were sectioned and stained with specific polyclonal antisera for AR. PSA and GCDFP-15. The ARu402 antiserum was a generous gift from Drs Michael J McPhaul. Carol M Wilson and Jean D Wilson (Department of Internal Medicine. University of Texas Southwestern Medical Center. Dallas. TX. USA). The PSA antiserum was supplied by Dako Corporation (Carpinteria. CA. USA). The GCDFP-15 antiserum was generously provided by Dr Darrow E Haagensen Jr (Methodist Hospital. Sacramento. CA. USA). The primary antibody reactions were incubated at 4°C overnight. the AR antiserum was diluted 1:500 in blocking solution (Hall et al. 1996): PSA. 1:1000 and GCDFP-15. 1:5000 (Mazoujian et al. 1983). AR was detected following microwave retrieval. as reported previously (Hall et al. 1996). PSA was detected in breast tumours following trypsin digestion: 0.1% w/v trypsin (Difco Laboratories. Detroit. MI. USA) and 0.1% $CaCl_2$ in Tris-buffered saline (0.005 M Tris-HCl. 0.145 M NaCl. pH 7.6). at 37°C for 30 min. Vectastain ABC kit (Vector Labs. Burlingame. CA. USA) and secondary anti-rabbit antibody (Vector Labs) were used routinely. Positive immunoreactivity was detected with the chromogen 3′3′-diaminobenzidine tetrahydrochloride (DAB: Sigma. St Louis. MO. USA) and negative cells were counterstained with weak Lillie Mayer's haematoxylin. Prostate tissues were used as AR- and PSA-positive controls. Normal rabbit immunoglobulin (Dako) was used as the negative primary antibody control. The specificity of PSA immunoreactivity in breast tumours was demonstrated by preabsorption (overnight at 4°C) of the antiserum with a 30-fold excess of purified PSA protein (Chemicon. Temecula. CA. USA). AR. PSA and GCDFP-15 immunoreactivity was quantified using video image analysis (VIA) (Hall et al. 1996). The percentage of immunopositive cells and the mean integrated optical density (MIOD). which measures the concentration of positive staining. were recorded.

### Statistical analysis

Relationships between AR. ER. PR. PSA. GCDFP-15. age. tumour grade and nodal status were analysed using chi-squared tests and logistic regression analysis.

### RESULTS

### AR, PSA and GCDFP-15 immunohistochemistry

AR immunoreactivity was localized in tumour cell nuclei and was heterogeneous. with both positively and negatively stained cells evident within breast tumours (Figure 1A). GCDFP-15 immunoreactivity was found in the cytoplasm of tumour cells and was heterogeneous. with focal staining in some tumours. whereas other tumours contained a high percentage of positively stained tumour cells (Figure 1B). Immunoreactive PSA was also localized in the cytoplasm of tumour cells (Figure 1C) and was present extracellularly in ductal luminae. Only a few breast tumours showed widespread staining for PSA (Figure 1C). whereas the majority of PSA-positive tumours showed focal PSA immunoreactivity (Figure 1D and E). The specificity of staining for PSA in breast tumours was demonstrated by preabsorption of the polyclonal antiserum with PSA protein (Figure 1E and F).

### Relationships between tumour variables

The highest incidence of immunoreactivity observed for the different variables examined (i.e. ER. PR. AR. PSA and GCDFP-15) was 85% (61/72) for the AR (Figure 2). AR immunoreactivity was significantly associated with the presence of both ER ($P$ < 0.05) and PR ($P$ < 0.01) in primary breast tumours (Table 1). ER and PR positivity were also significantly associated ($P$ < 0.001). The presence of AR in breast tumours was significantly

© Cancer Research Campaign 1998

**Figure 2** The percentage of primary breast tumours ($n$ = 72) with positive immunoreactivity for ER, PR, AR, PSA and GCDFP-15 respectively

**Table 4** Relationships between PSA or GCDFP-15 and ER and PR status in 72 primary breast tumours

| | ER | | PR | |
|---|---|---|---|---|
| | +ve | –ve | +ve | –ve |
| PSA +ve | 34[a] | 10 | 36[b] | 8 |
| –ve | 18 | 10 | 21 | 7 |
| GCDFP-15 +ve | 35[c] | 13 | 37[d] | 11 |
| –ve | 17 | 7 | 20 | 4 |

[a]$\chi^2$ = 1.44, $P$ = 0.23. [b]$\chi^2$ = 0.48, $P$ = 0.49. [c]$\chi^2$ = 0.04, $P$ = 0.85. [d]$\chi^2$ = 0.38, $P$ = 0.54.

**Table 5** Logistic regression analysis of tumour variables with PSA and GCDFP-15

| Tumour variable | Odds ratio (95% CI[a]) | | Odds ratio (95% CI) | |
|---|---|---|---|---|
| | PSA | | GCDFP-15 | |
| Age | 1.0 | (1.0–1.0) | 1.0 | (1.0–1.0) |
| AR status[b] | 23.9 | (2.8–200.6) | 4.5 | (1.2–17.5) |
| ER status | 1.9 | (0.7–5.4) | 1.1 | (0.4–3.3) |
| PR status | 1.5 | (0.5–4.7) | 0.7 | (0.2–2.4) |
| Grade I[c] | 1.0 | | 1.0 | |
| Grade II | 0.3 | (0.1–1.7) | 0.5 | (0.1–2.2) |
| Grade III | 0.1 | (0.1–0.7) | 0.4 | (0.9–1.7) |
| Nodal status | 0.5 | (0.2–1.5) | 0.8 | (0.3–2.4) |

[a]Confidence intervals (CI) that include one indicate a non-significant effect. [b]The odds ratios for an AR-positive tumour being positive for PSA or GCDFP-15, respectively, were significantly greater than one. [c]Using Grade I as the referent category, the odds ratio for a grade III tumour being PSA positive was significantly less than (one-tenth) the odds of a grade I tumour being PSA positive.

associated with both PSA ($P$ < 0.001) and GCDFP-15 ($P$ < 0.05) immunostaining (Table 2), with the majority of PSA-positive (98%, 43/44) and GCDFP-15-positive (92%, 44/48) tumours being AR positive. There was a significant association between the presence of PSA and GCDFP-15 ($P$ < 0.001), with 86% (38/44) of PSA-positive tumours also positive for GCDFP-15. PSA and GCDFP-15 were co-expressed in 62% (38/61) of AR-positive breast tumours, with a higher proportion of lower grade (i.e. grades I and II) primary breast tumours being PSA positive compared with grade III tumours (Table 3; $P$ < 0.05). No significant associations were found between PSA and ER status, PSA and PR status, GCDFP-15 and ER status or GCDFP-15 and PR status in the breast tumours (Table 4). AR immunoreactivity was the only significant factor predicting both PSA- and GCDFP-15-positive staining in breast tumours (Table 5). AR-positive tumours were 24 and 4.5 times more likely to be PSA and GCDFP-15 positive respectively. Another factor significantly associated with PSA positivity was tumour grade; grade III tumours were nine times more likely to be PSA negative than grade I tumours (Table 5). Age, ER and PR status of the tumour and axillary lymph node involvement were not significantly associated with PSA or GCDFP-15 immunoreactivity in primary breast tumours (i.e. odds ratios included 1.0).

### DISCUSSION

AR immunoreactivity was present in a high proportion (85%) of primary breast tumours and AR was frequently co-expressed with ER and PR, as reported in previous studies (Lea et al, 1989; Kuenen-Boumeester et al, 1992; Isola, 1993; Hall et al, 1996). This study identified for the first time a highly significant association between PSA and GCDFP-15 expression in breast tumours, with PSA and GCDFP-15 being co-expressed in 62% of AR-positive breast tumours. The expression of both PSA and GCDFP-15 was dependent upon the presence of AR in primary breast tumours, but was unrelated to patient age, ER and PR status of the primary tumour and nodal status.

GCDFP-15 immunoreactivity was detected in 67% (48/72) of primary breast tumours in this study. Although previous studies indicate that approximately 50% of all breast carcinomas contain GCDFP-15 protein (Haagensen Jr, 1991), there is considerable variation in the reported incidence of GCDFP-15. Variation between immunohistochemical studies has also been attributed to differences in sensitivity between GCDFP-15 antisera (cited in Pagani et al, 1994). While the function of PSA and GCDFP-15 in breast tumours is not known, these proteins may interact to modulate tumour invasion of the extracellular matrix.

The incidence of PSA-positive tumours in the present study (i.e. 61%) is higher than first reported (i.e. 30%) for PSA measured by enzyme immunoassay in breast tumour cytosolic extracts (Diamandis et al, 1994). The incidence of PSA-positive tumours determined in our study, however, is similar to that (i.e. 70%) reported by Ferguson et al (1996), who used an ultrasensitive immunoassay with a lower limit of detection (< 1 ng $l^{-1}$). Our study is also in agreement with a recent immunohistochemical study of PSA using the same polyclonal antibody (Howarth et al, 1997). In that study 62% (13/21) of infiltrating ductal carcinomas were at least faintly positive for PSA. In contrast, Wu et al (1995) reported a 15% incidence of PSA-positive breast tumours. The lower incidence in Wu's study was attributed to a lack of PSA detection in frozen cytosols as a result of the instability of the PSA protein.

Other studies, including those from our laboratory (Abrahamsson et al. 1988; Aspinall et al. 1995; Zhang et al. 1998), have demonstrated PSA reactivity in virtually all non-malignant prostates and early-stage prostate tumours examined. In contrast, the amounts of PSA protein in primary breast tumours, i.e. the percentage of immunopositive cells and the concentration of PSA, appear to be appreciably lower. This may reflect tissue-specific differences in the regulation of PSA in breast versus prostate. Despite earlier studies that demonstrate significant associations between PSA positivity and either ER (Yu et al. 1995*b*) or PR (Yu et al. 1994), or both ER and PR positivity in breast tumour cytosols (Ferguson et al. 1996), no significant associations were found between PSA and ER or PR status in the present study (Table 4). Importantly, one of the previous studies identified that PSA positivity was significantly associated with PR and not with ER in subsets of breast tumours (viz. ER + PR +; ER – PR +) (Yu et al. 1994). The failure to detect a direct association between PSA and PR in our study was possibly due to the relatively small number of cases (72) studied. In our study, PSA positivity was associated with breast tumours of lower histological grades. Loss of PSA expression therefore is likely to be associated with a poorer outcome, as recently found by Yu et al (1997). Interestingly, there is a similar reduction in PSA immunoreactivity in cases of prostate cancer compared with benign prostatic hyperplasia, which has more homogeneous PSA staining and a greater percentage of PSA-positive cells (Abrahamsson et al. 1988; Aspinall et al. 1995). Therefore, in both breast and prostate cancer, it appears that loss of PSA protein is associated with de-differentiation during tumour development and progression.

In a previous study, we demonstrated that the level of AR in primary breast tumours is associated with response to second-line therapy with the synthetic progestin medroxyprogesterone acetate (Birrell et al. 1995), indicating the potential usefulness of AR as a prognostic marker. In the present study, we have shown that, although AR, ER and PR are co-expressed in breast tumours, PSA and GCDFP-15 positivity is significantly associated with AR alone and not determined by ER or PR status of the primary tumour. The data from this and previous studies suggest that the expression of PSA and GCDFP-15 may be dependent on a functional AR pathway. This study also demonstrates that immunoreactive PSA, like GCDFP-15 (Haagensen Jr. 1991), is strongly associated with the presence of AR in primary breast tumours, suggesting that both PSA and GCDFP-15 are androgen-regulated in breast tumours in vivo.

In conclusion, although it has been suggested that PSA and GCDFP-15 may facilitate tumour invasion, the evidence presented in this study clearly supports their association with a well-differentiated phenotype in breast cancer and, therefore, better prognosis.

### ABBREVIATION

AR, androgen receptor; DAB, 3′3′-diaminobenzidine tetrahydrochloride; DCIS, ductal carcinoma in situ, ER, oestrogen receptor; GCDFP-15, gross cystic disease fluid protein of mol. wt 15 kDa; MIOD, mean integrated optical density; PR, progesterone receptor; PSA, prostate-specific antigen; VIA, video image analysis.

### ACKNOWLEDGEMENTS

We wish to thank Ms Coralie Vella for technical assistance with video image analysis measurements and Ms Lynne Giles, Computing Services, The Flinders University of South Australia, for performing the statistical analysis. This work was supported by the Kathleen Cuningham Foundation for Breast Cancer Research, the Anti-Cancer Foundation of the Universities of South Australia and the National Health and Medical Research Council of Australia.

### REFERENCES

Abrahamsson P-A, Lilja H, Falkmer S and Wadström LB (1988) Immunohistochemical distribution of the three predominant secretory proteins in the parenchyma of hyperplastic and neoplastic prostate glands. *Prostate* **12**: 39–46

Aspinall JO, Bentel JM, Horsfall DJ, Haagensen DE, Marshall VR and Tilley WD (1995) Differential expression of apolipoprotein-D and prostate specific antigen in benign and malignant prostate tissues. *J Urol* **154**: 622–628

Autiero M, Cammarota G, Friedlein A, Zulauf M, Chiapetta G, Dragone V and Guardiola J (1995) A 17-kDa CD4-binding glycoprotein present in human seminal plasma and in breast tumor cells. *Eur J Immunol* **25**: 1461–1464

Birrell SN, Roder DM, Horsfall DJ, Bentel JM and Tilley WD (1995) Medroxyprogesterone acetate therapy in advanced breast cancer: the predictive value of androgen receptor expression. *J Clin Oncol* **13**: 1572–1577

Clements JA (1994) The human kallikrein gene family: a diversity of expression and function. *Mol Cell Endocrinol* **99**: C1–C6

Clements J and Mukhtar A (1994) Glandular kallikreins and prostate-specific antigen are expressed in the human endometrium. *J Clin Endocrinol Metab* **78**: 1536–1539

Clements JA, Mukhtar A, Verity K, Pullar M, McNeill P, Cummins J and Fuller PJ (1996) Kallikrein gene expression in human pituitary tissues. *Clin Endocrinol* **44**: 223–231

Clements JA, Ward G, Kaushal A, Hii S-I, Kennett C and Nicol D (1997) A prostate specific antigen (PSA)-like protein associated with renal cell carcinoma in women. *Clin Cancer Res* **3**: 1427–1431

Cohen P, Graves HCB, Peehl DM, Kamarei M, Giudice LC and Rosenfeld RG (1992) Prostate-specific antigen (PSA) is an insulin-like growth factor binding protein-3 protease found in seminal plasma. *J Clin Endocrinol Metab* **75**: 1046–1053

Diamandis EP and Yu H (1995) Editorial: new biological functions of prostate-specific antigen? *J Clin Endocrinol Metab* **80**: 1515–1517

Diamandis EP and Yu H (1995) Nonprostatic sources of prostate-specific antigen. *Urol Clin North Am* **24**: 275–282

Diamandis EP, Yu H and Sutherland DJA (1994) Detection of prostate-specific antigen immunoreactivity in breast tumors. *Breast Cancer Res Treat* **32**: 301–310

Ferguson RA, Yu H, Kalyvas M, Zammit S and Diamandis EP (1996) Ultrasensitive detection of prostate-specific antigen by a time-resolved immunofluorometric assay and the Immulite® immunochemiluminescent third-generation assay: potential applications in prostate and breast cancers. *Clin Chem* **42**: 675–684

Haagensen DE, Kelly D and Bodian CA (1997) GCDFP-15 blood levels for stratification of risk of breast cancer development in women with active breast gross cystic disease. *Breast* **6**: 113–119

Haagensen Jr DE (1991) Is cystic disease related to breast cancer? *Am J Surg Pathol* **15**: 687–694

Hall RE, Aspinall JO, Horsfall DJ, Birrell SN, Bentel JM, Sutherland RL and Tilley WD (1996) Expression of the androgen receptor and an androgen-responsive protein, apolipoprotein D, in human breast cancer. *Br J Cancer* **74**: 1175–1180

Henttu P, Liao S and Vihko P (1992) Androgens up-regulate the human prostate-specific antigen messenger ribonucleic acid (mRNA), but down-regulate the prostatic acid phosphatase mRNA in the LNCaP cell line. *Endocrinology* **130**: 766–772

Howarth DJC, Aronson IB and Diamandis EP (1997) Immunohistochemical localization of prostate-specific antigen in benign and malignant breast tissues. *Br J Cancer* **75**: 1646–1651

Isola JJ (1993) Immunohistochemical demonstration of androgen receptor in breast cancer and its relationship to other prognostic factors. *J Pathol* **170**: 31–35

Iwamura M, Hellman J, Cockett ATK, Lilja H and Gershagen S (1996) Alteration of the hormonal bioactivity of parathyroid hormone-related protein (PTHrP) as a result of limited proteolysis by prostate-specific antigen. *Urology* **48**: 317–325

Kuenen-Boumeester V, van der Kwast TH, van Putten WLJ, Claassen C, van Ooijen B and Henzen-Logmans SC (1992) Immunohistochemical determination of androgen receptors in relation to oestrogen and progesterone receptors in female breast cancer. *Int J Cancer* **52**: 581–584

Lea OA, Kvinnsland S and Thorsen T (1989) Improved measurement of androgen receptors in human breast cancer. *Cancer Res* **49**: 7162–7167

Lilja H (1985) A kallikrein-like serine protease in prostatic fluid cleaves the predominant seminal vesicle protein. *J Clin Invest* **76**: 1899–1903

© Cancer Research Campaign 1998

Lilja H, Oldbring J, Rannevik G and Laurell C-B (1987) Seminal vesicle-secreted proteins and their reactions during gelation and liquefaction of human semen. *J Clin Invest* **80**: 281–285

Mannello F, Bocchiotti GD, Bianchi G, Marcheggiani F and Gazzanelli G (1996) Quantitation of prostate-specific antigen immunoreactivity in human breast cyst fluids. *Breast Cancer Res Treat* **38**: 247–252

Mazoujian G, Pinkus GS, Davis S and Haagensen Jr DE (1983) Immunohistochemistry of a gross cystic disease fluid protein (GCDFP-15) of the breast – a marker of apocrine epithelium and breast carcinomas with apocrine features. *Am J Pathol* **110**: 105–112

Melegos DN, Yu H and Diamandis EP (1996) Prostaglandin $D_2$ synthase: a component of human amniotic fluid and its association with fetal abnormalities. *Clin Chem* **42**: 1042–1050

Murphy LC, Lee-Wing M, Goldenberg GJ and Shiu RPC (1987) Expression of the gene encoding a prolactin-inducible protein by human breast cancers *in vivo*: correlation with steroid receptor status. *Cancer Res* **47**: 4160–4164

Myal Y, Robinson DB, Iwasiow B, Tsuyuki D, Wong P and Shiu RPC (1991) The prolactin-inducible protein (*PIP*/GCDFP-15) gene: cloning, structure and regulation. *Mol Cell Endocrinol* **80**: 165–175

Oesterling JE (1991) Prostate specific antigen: a critical assessment of the most useful tumor marker for adenocarcinoma of the prostate. *J Urol* **145**: 907–923

Pagani A, Sapino A, Eusebi V, Bergnolo P and Bussolati G (1994) PIP/GCDFP-15 gene expression and apocrine differentiation in carcinomas of the breast. *Virchows Archiv* **425**: 459–465

Papotti M, Paties C, Peveri V, Moscuzza L and Bussolati G (1989) Immunocytochemical detection of prostate-specific antigen (PSA) in skin adnexal and breast tissues and tumors. *Bas Appl Histochem* **33**: 25–29

Pummer K, Wirnsberger G, Pürstner P, Stettner H and Wandschneider G (1992) False positive prostate specific antigen values in the sera of women with renal cell carcinoma. *J Urol* **148**: 21–23

Schenkels LCPM, Schaller J, Walgreen-Weterings E, Schadee-Eestermans IL, Veerman ECI and Amerongen AVN (1994) Identity of human extra parotid glycoprotein (EP-GP) with secretory actin binding protein (SABP) and its biological properties. *Biol Chem Hoppe-Seyler* **375**: 609–615

Webber MM, Waghray A and Bello D (1995) Prostate-specific antigen, a serine protease, facilitates human prostate cancer cell invasion. *Clin Cancer Res* **1**: 1089–1094

Wu JT, Zhang P, Astill ME, Wilson LW, Lyons BW, Wu LL and Stephenson R (1995) PSA immunoreactivity detected in LNCaP cell medium, breast tumor cytosol, and female serum. *J Clin Lab Anal* **9**: 243–251

Yu H, Diamandis EP and Sutherland DJA (1994) Immunoreactive prostate-specific antigen levels in female and male breast tumors and its association with steroid hormone receptors and patient age. *Clin Biochem* **27**: 75–79

Yu H, Diamandis EP, Levesque M, Asa SL, Monne M and Croce CM (1995*a*) Expression of the prostate-specific antigen gene by a primary ovarian carcinoma. *Cancer Res* **55**: 1603–1606

Yu H, Giai M, Diamandis EP, Katsaros D, Sutherland D, Levesque MA, Roagna R, Ponzone R and Sismondi P (1995*b*) Prostate-specific antigen is a new favorable prognostic indicator for women with breast cancer. *Cancer Res* **55**: 2104–2110

Yu H, Levesque MA, Clark GM and Diamandis EP (1997) *Prognostic Value of PSA in Breast Cancer: a Large US Cohort Study*. Am Assoc Cancer Res, San Diego, pp. 437

Zarghami N, Grass L and Diamandis EP (1997) Steroid hormone regulation of prostate-specific antigen gene expression in breast cancer. *Br J Cancer* **75**: 579–588

Zhang SXD, Bentel JM, Ricciardelli C, Horsfall DJ, Haagensen DE, Marshall VR and Tilley WD (1998) Immunolocalization of apolipoprotein D, androgen receptor and prostate specific antigen in early stage prostate cancers. *J Urol* **159**: 548–554

© Cancer Research Campaign 1998